# HEALTH CARE USE IN DIVERSE OLDER ADULT POPULATIONS WITH ALZHEIMER’S DISEASE AND RELATED DEMENTIAS

**DOI:** 10.1093/geroni/igae098.3094

**Published:** 2024-12-31

**Authors:** Gee Kim

**Affiliations:** FAIR Health, New York, New York, United States

## Abstract

Average medical costs for people aged 65 and older with a diagnosis of Alzheimer’s disease and related dementias (ADRD) are far higher than for people in the same age range without an ADRD diagnosis. Prior research shows the incidence of ADRD is significantly higher for Black and Hispanic populations than for non-Hispanic white and Asian populations. It is unclear, however, whether there are significant racial and ethnic disparities in the overall utilization and cost of care among patients with ADRD. An independent nonprofit organization, with the nation’s largest collection of private healthcare claims, studied the relationship between imputed race and ethnicity and overall healthcare utilization and spending in over one million patients aged 65 and older with a diagnosis of ADRD. Among the preliminary findings: In comparison with patients in other racial/ethnic groups, Black patients with ADRD had (1) on average poorer health, as measured by a higher average number of chronic conditions and a higher Hierarchical Conditions Category (HCC) score; (2) more intensive healthcare utilization, i.e., on average more admissions, longer admissions, more total days spent in the hospital, more claims filed, more distinct procedures received and more distinct providers seen; and (3) higher spending even after controlling for race/ethnicity-related HCC scores. These findings suggest race/ethnicity influences healthcare utilization and spending directly and indirectly for patients with ADRD. Results can be used to inform healthcare research, clinical guidelines, care delivery and benefits for older people with ADRD, as well as inform health equity approaches among minority and diverse populations.

